# Challenges of linking to routine healthcare records in UK Biobank

**DOI:** 10.1186/1745-6215-16-S2-O68

**Published:** 2015-11-16

**Authors:** Ligia Adamska, Naomi Allen, Robin Flaig, Cathie Sudlow, Michael Lay, Martin Landray

**Affiliations:** 1 University of Oxford, Oxford, UK; 2 UK Biobank, Stockport, UK; 3 University of Edinburgh, Edinburgh, UK

## 

Increased availability of electronic healthcare records (EHR) has transformed how health research is conducted in the UK by enabling linkages between various health-related datasets.

UK Biobank is a prospective cohort study of 500,000 men and women aged 40-69 years recruited throughout the UK between 2006 and 2010. To follow participants' health over time, UK Biobank has linked to national death and cancer registries and hospital admissions data, with linkage to primary care data under development. This exercise involves linking to separate data providers, determining which data-fields are of most value to research, mapping changes in clinical coding systems over time, and distinguishing which data can be standardised from those that should remain specific to the dataset of origin, before integration into a single dataset amenable to analysis by external researchers.

Data linkage for a national cohort poses several challenges including different regulatory processes across each of the devolved data providers, as well as differences in matching algorithms, data formats and coding schema, in addition to the sheer volume of data to be processed. One of the biggest challenges is defining rules for handling data ambiguities whilst preserving data integrity and provenance. UK Biobank is the first study to map hospital episode records across England, Scotland and Wales. These datasets vary in terms of content, data quality, geographical and temporal coverage and considerable expertise is required to integrate, document and present these data in an accessible way to researchers.

